# New Literary Journals

**Published:** 1866-02

**Authors:** 


### New Literary Journals.

We have recently had placed upon our table several new literary journals, all of which are very welcome visitors. Ticknor & Fields, the Boston publishers, who have done so much to gratify the literary taste of the country in publishing the "Atlantic Monthly," now send us the "Every Saturday," which, as its name indicates, is to be published weekly. It is a journal of choice reading, selected from the foreign current literature, and will no doubt be not only entertaining and instructive, but as proposed by the publishers will be an "interesting and valuable reflex of foreign periodical literature of the better class."

The numbers which we have received fully warrant the assurance that *Every Saturday* will be one of the most entertaining of peri odicals, and will be an attraction to all intelligent and cultivated readers.

*American Educational Monthly*, devoted to popular instruction and literature, has also been received, and its contents perused. We find it full of instructive and entertaining reading, richly meriting the reputation it has gained as a popular literary journal. It is published by Schermerhorn, Bancroft & Co., 130 Grand St., New York, for $1.50 per year, thus making it within reach of all. From the contents of the numbers we have received, we are greatly prejudiced in its favor. It seems to be the governing purpose to instruct, and at the same time entertain. We are much obliged to the publishers for the courtesy extended in sending this magazine.

